# A Rare Case of Adamantinomatous Craniopharyngioma in an Adult

**DOI:** 10.7759/cureus.30000

**Published:** 2022-10-06

**Authors:** Emilee A Carpenter, Omari Christie, Christine Fuller, Kavya Mirchia

**Affiliations:** 1 Radiology, State University of New York Upstate Medical University, Syracuse, USA; 2 Diagnostic Radiology, State University of New York Upstate Medical University, Syracuse, USA; 3 Pathology, State University of New York Upstate Medical University, Syracuse, USA; 4 Neuroradiology, State University of New York Upstate Medical University, Syracuse, USA

**Keywords:** pituitary, radiology of craniopharyngioma, sellar tumor, adult brain tumor, adamantinomatous craniopharyngioma

## Abstract

Craniopharyngiomas represent a rare group of intracranial tumors that often arise in the sellar/suprasellar region of the brain. Adamantinomatous craniopharyngioma is significantly more common than papillary craniopharyngioma. The former most often arises in children whereas the papillary craniopharyngioma is mainly limited to adults. We present the case of a 34-year-old female with visual disturbances and other vague complaints who was found to have a large lobulated sellar mass on neuroimaging studies. She was subsequently diagnosed with an adamantinomatous craniopharyngioma after undergoing transsphenoidal resection. We discuss the patient’s clinical, radiological, and pathological findings in correlation with the current literature and recommendations regarding this type of tumor. Given that adamantinomatous craniopharyngioma rarely presents in adulthood, especially in middle-aged adults, this case is considered rare, and we hope to increase awareness to include adamantinomatous craniopharyngioma in the differential diagnosis for sellar lesions in this age group.

## Introduction

Craniopharyngiomas are rare, benign tumors that arise from the embryonic remnants of Rathke’s pouch. They include two distinct tumor entities, namely, adamantinomatous craniopharyngiomas (ACP) and papillary craniopharyngiomas (PCP) [1]. Typically, the former occurs in childhood whereas papillary craniopharyngioma arises almost exclusively in adults [1,2]. As a group, craniopharyngiomas represent approximately 2-5% of all intracranial tumors [3] and follow a bimodal age distribution. Although there is variation in the literature, the age of occurrence is around 5-14 and 40-70 years of age [4-6].

The typical manifestations of craniopharyngioma are a result of mass effects on nearby structures and include headache, vision changes, and numerous endocrine disturbances. The World Health Organization (WHO) Classification of CNS Tumours considers craniopharyngioma (both ACP and PCP) as CNS WHO grade 1 [7], although its propensity to invade local structures including the pituitary, hypothalamus, and blood vessels can increase its associated mortality [8]. Nevertheless, it has a survival rate of over 85% after 20 years, following treatment [7]. The mainstay of treatment is surgical resection but can also include radiation or chemotherapy for patients undergoing partial resection or who are not surgical candidates [8]. There exist limited targeted therapies for the adamantinomatous subtype; however, gene mutations have been identified that could eventually provide a target for treatment.

We describe a rare case of ACP in a middle-aged adult. We discuss the radiological, pathological, and clinical importance of this case while supporting the current literature and hope to increase awareness and knowledge for clinicians encountering this entity in the future.

## Case presentation

A 34-year-old female presented to the emergency room upon recommendations from her ophthalmologist due to ongoing (over several years) and now worsening blurry vision. Two weeks prior, she had numerous teeth extracted and noticed an acute worsening of her vision. Chronic ongoing issues included amenorrhea around age 19, weight loss of 15 pounds (2.3 kg) over several months (attributed to lack of eating secondary to dental discomfort), dizziness upon standing, frequent severe, throbbing, mostly right-sided headaches, and an achy pain over her forehead for two to three months, unique from the previously described headaches, unresponsive to any medications.

Her medical history was significant for anxiety and migraines, treated with daily venlafaxine. Upon review of systems, she denied recent illness, shortness of breath, nausea, vomiting, fever, or chills. The physical examination was unrevealing. Ophthalmology consultation identified significant visual field restrictions in the left superior temporal, inferior temporal, and superior nasal areas. Extraocular movements were intact bilaterally. Visual acuity was found to be 20/200 on the right. The left side was evaluated with a hand motion. All other visual tests were within normal limits.

**Figure 1:**
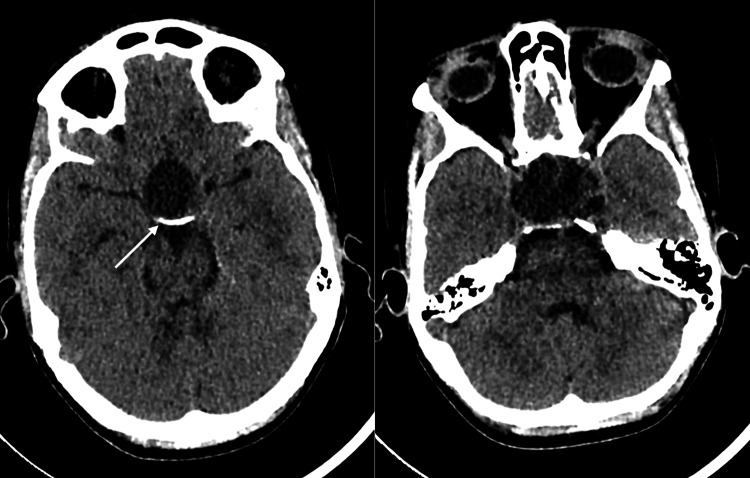
Axial non-contrast computed tomography of the head. Large cystic sellar/suprasellar mass with peripheral calcifications (arrow) and causing excessive expansion of the sella.

**Figure 2:**
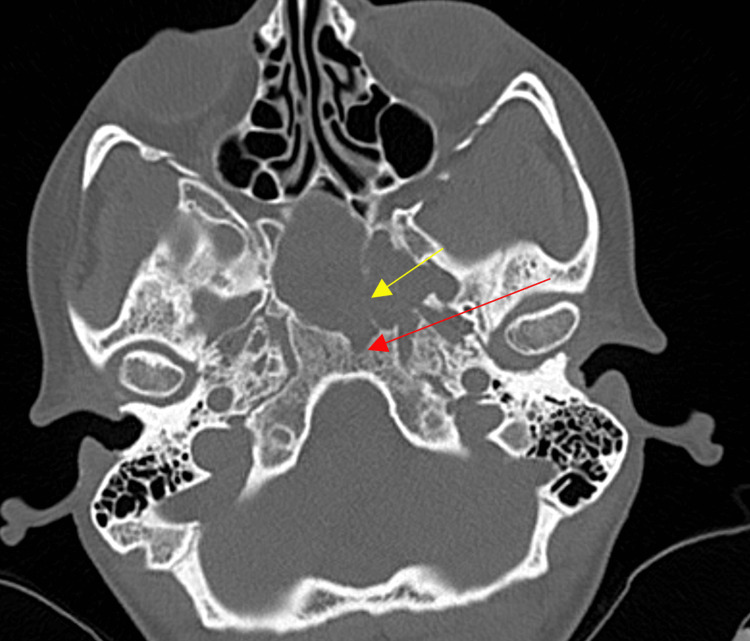
Axial non-contrast computed tomography of the head bone window. Bone window showing compression and partial erosion of the left sphenoid bone (yellow arrow) and the clivus (red arrow).

**Figure 3:**
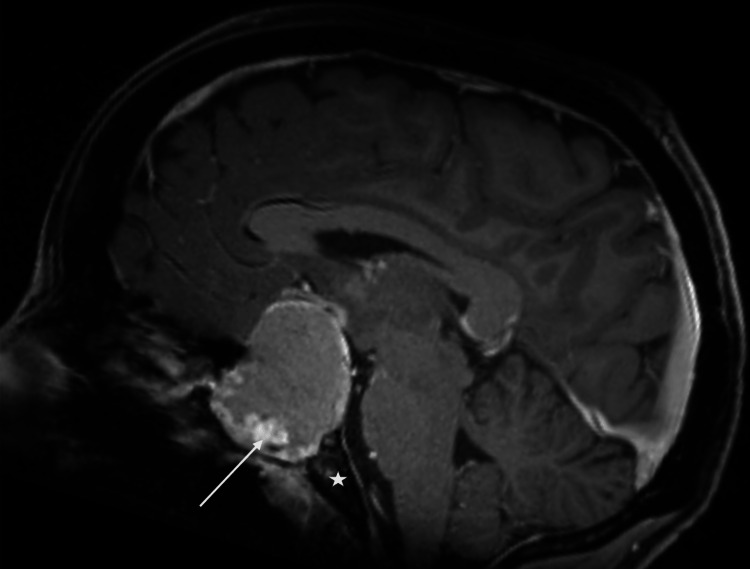
Sagittal T1-weighted post-contrast magnetic resonance imaging. Sagittal T1 post-contrast magnetic resonance image demonstrating a suprasellar lesion with enhancing nodular components (arrow). Clivus is represented by the star.

**Figure 4:**
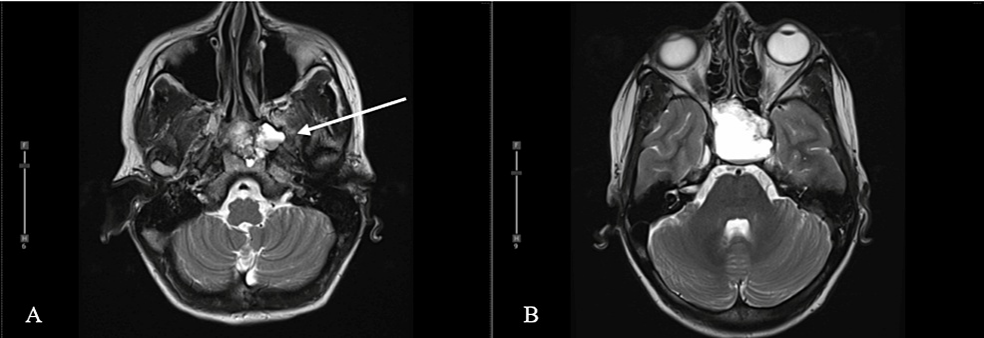
Axial T2-weighted non-contrast magnetic resonance images. Axial T2-weighted non-contrast magnetic resonance images obtained at the level of the masticator space (A) and at the level of the globes (B). Image A demonstrates the asymmetric extension of the lesion through the left sphenoid bone and into the masticator space (arrow). In image B, there is a deformity of the sphenoid sinuses secondary to mass effect from the lesion, greater on the left.

**Figure 5:**
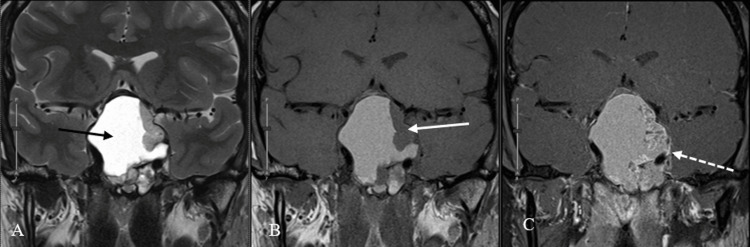
Coronal magnetic resonance images of varying sequences. Coronal T2 (A), T1 (B), and T1 post-contrast (C) magnetic resonance images. This lesion is composed of cystic (black arrow) and solid (white arrow) components evidenced by non-enhancing central T2 hyperintensity and enhancement within the peripheral nodular components (dotted white arrow).

Initial computed tomography CT is demonstrated below (Figures 1, 2). Magnetic resonance imaging (MRI) of the brain with and without contrast revealed a lobulated cystic and solid, contrast-enhancing seller mass measuring approximately 38 × 38 × 47 mm in the transverse, anteroposterior, and craniocaudal dimensions, respectively (Figure 3). There were cystic components, which deformed the sphenoid sinus, and partially extended through the sphenoid bone, more so on the left (Figure 4). The mass was predominantly T2 hyperintense with peripheral regions of nodular enhancement (Figures 5A-5C). Inferiorly, there were foci of susceptibility-weighted artifact within the solid component of the lesion likely representing calcification. The lesion did not significantly restrict diffusion.

Endocrine and neurosurgery teams were consulted. The patient began taking hydrocortisone at physiologic doses which increased to stress doses at the time of surgery due to a low morning cortisol level. Additional endocrine testing revealed elevated prolactin and low insulin-like growth factor 1. Thyroid-stimulating hormone, luteinizing hormone, and follicle-stimulating hormone levels were within normal limits. The patient underwent transsphenoidal resection of the tumor. Intraoperatively, the tumor was found to be heterogeneous with both soft and firm areas; it completely filled the sphenoid sinus, and invaded the cavernous sinus walls, predominantly on the left side. The compressed pituitary gland and stalk were identified and preserved during the surgery. Postoperatively, the patient reported subjective improvement in her vision the same day.

**Figure 6:**
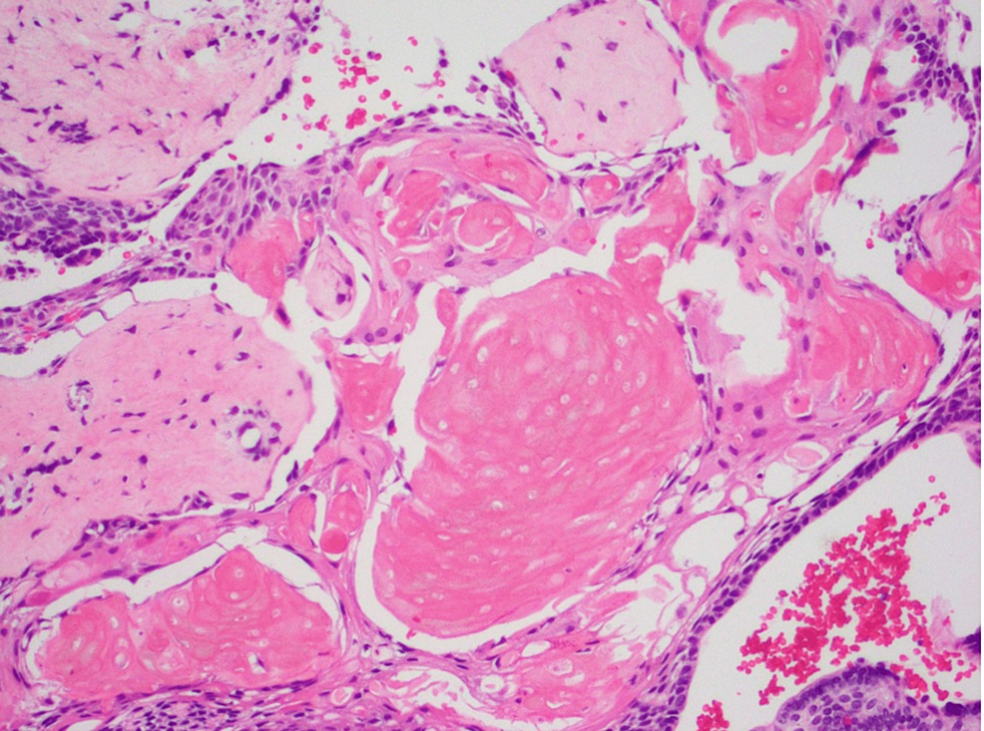
Hemosiderin and eosin staining of surgical pathology sellar mass, fresh section. Extensive wet keratin is present in this adamantinomatous craniopharyngioma. Palisading epithelium is present in the lower right.

**Figure 7:**
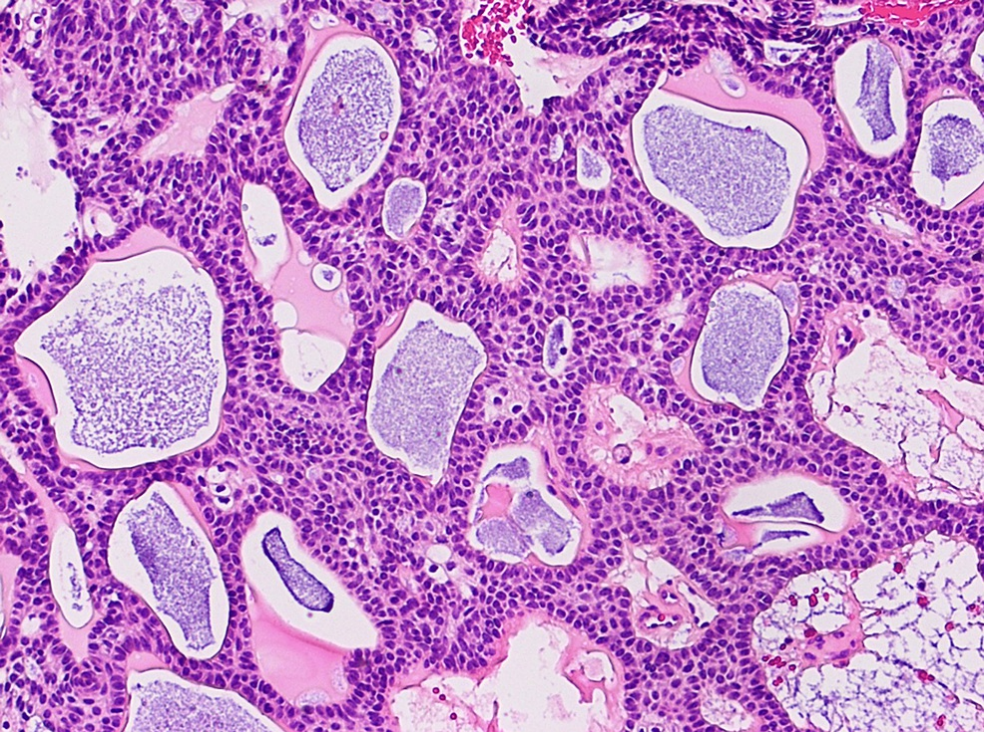
Hemosiderin and eosin stain of surgical pathology, sellar mass. Multicystic architecture with palisading of epithelium around mucin-filled cystic spaces.

Pathologic evaluation of the tissue specimen revealed an adamantinomatous craniopharyngioma with typical basaloid palisaded epithelium, stellate reticulum, areas of wet keratin, and multiple cystic spaces, some containing mucinous material (Figures 6, 7). Microcalcifications were also detected. The patient developed transient diabetes insipidus postoperatively and received subcutaneous desmopressin (DDAVP) in response. The patient was discharged from the hospital a few days after surgery. She did require thyroid replacement therapy and is currently on daily 50 µg levothyroxine. A vision assessment a few months after the surgery showed a stable examination; however, visual acuity worsened in the right eye and improved in the left eye.

## Discussion

ACPs are extremely rare in adults, with most adult cases presenting over 50 years of age [9,10]. As the distribution of these tumors follows a bimodal curve, the age at diagnosis for our patient makes this case even more unique.

In an epidemiological study in the United States, PCPs were diagnosed in 5.5% of patients aged 0-29 years old, 30.6% in 30-59 years old, and 30.4% in patients over 60. PCP was found to be more common in adults; however, ACP was significantly more common overall when compared to its papillary counterpart. In addition, the incidence of craniopharyngioma was found to be the greatest amongst Blacks followed by Whites, Asian or Pacific Islanders, and American Indian/Alaska natives. There was no difference in incidence between genders [5].

Craniopharyngiomas typically arise in the suprasellar area of the brain and vary in size. Tumors that have both a sellar and suprasellar component, as in our case, comprise around 53-75% of all cases [2]. There are rare instances in which craniopharyngiomas present outside of this region, including the cerebellopontine angle as a common ectopic site [11]. Our patient’s tumor was in the sellar and suprasellar areas, partially eroding the left sphenoid bone and cavernous sinus walls.

From a neuroimaging standpoint, the lobulated shape is a reliable feature in helping differentiate between ACP and PCP [12]. Lobulations were noted on our patient’s MR images.

Craniopharyngiomas often comprise cystic fluid to some degree. Therefore, their signal intensity on MRI can vary. Typically, solid portions of the tumor have high signal intensity on T1-weighted images. This is thought to be secondary to high protein content (motor oil cysts), hemorrhage, cholesterol, or a few calcifications [10]. According to a 2016 study, there seems to be a difference in T1 hyperintensity of ACPs based on the age of the patient. Fewer adult adamantinomatous tumors were bright on T1 whereas a higher percentage of pediatric cases showed high-intensity T1 signal. Additionally, there is a commonality between both subtypes to show cystic changes on T2-weighted images [13]. Our case depicted a few typical imaging findings of ACPs in an adult, usually seen in pediatric patients, namely, T2 hyperintense cystic component with T1 hyperintense motor-oil component and an enhancing peripheral solid component.

Calcifications are a defining feature of ACP and are more common in pediatric cases [13]. Calcifications can be easily identified on a CT scan. The CT scan performed for our patient prior to surgery demonstrated calcifications in the periphery of the mass in the sellar and suprasellar regions. Regions of calcification were also seen on susceptibility-weighted images on MRI.

The typical histopathological findings of the adamantinomatous craniopharyngioma include “squamous epithelium disposed in cords, nodules, and irregular trabeculae bordered by palisaded columnar epithelium” [2]. Our case also contained cystic structures with mucinous material, abundant wet keratin, and calcification, all features commonly present in this tumor type. In contradistinction, PCPs microscopically resemble squamous papillomas, composed of fibrovascular cores covered by well-differentiated, non-keratinizing stratified squamous epithelium.

The patient described numerous chronic abnormalities such as changes in menstruation, weight loss, dizziness, and headaches when she presented for evaluation. Although the etiology of these complaints had yet to be determined, the craniopharyngioma likely served as the underlying common source. Our patient also developed transient diabetes insipidus after her transsphenoidal resection which was successfully treated with DDAVP. She currently requires thyroid replacement therapy, a new medication addition after resection. A study done in 2019 found that the effect of the tumor on hypothalamic dysfunction, mass effect, and incidence of central diabetes insipidus was found to be lower in the adamantinomatous group when compared to PCP. This remained true whether the surgery had been completed or not. However, postsurgery hypothalamic and pituitary dysfunction and incidence of central diabetes insipidus were increased significantly for both tumor types. More than half of the patients typically complained of vision impairment at diagnosis, and 41-48% reported postoperative improvement in vision [14], as is true in the current case.

Although there have yet to be prospective treatment trials, surgical resection is typically the first-line treatment of craniopharyngiomas. Given that ACP tends to adhere more to its surroundings and can surround blood vessels, this poses a neurosurgical challenge and can limit cases attaining gross total resection [15]. Adjunct treatment with radiotherapy or chemotherapy may need to be employed and should be discussed, accounting for individual patient clinical scenarios.

Studies have shown that recurrence rates of craniopharyngiomas are highly dependent on the extent of surgical resection; however, tumors invading the hypothalamus are associated with significant morbidity. There was no difference in five- and ten-year survival rates between patients who have undergone gross total resection versus subtotal resection of the tumors with adjuvant therapy. However, patients who underwent subtotal resection without adjuvant therapy had significantly higher recurrence rates when compared to subtotal resection with advent therapy [6].

There have been numerous studies evaluating the genetic alterations in craniopharyngioma that could be crucial to future treatment strategies. *β-catenin* gene mutations have been documented in more than 70% of ACPs [2]. *BRAF V600E* mutations have been found in the majority of the PCP cases but have not been associated with the adamantinomatous subtype [16], while *CTNNB1 *mutations were found in 75-96% of the adamantinomatous subtype [10]. While surgery is the mainstay of treatment, radiation and/or chemotherapy may be appropriate. More targeted therapies aimed at immune markers such as interleukin 6 and checkpoint inhibitors seem to be a therapeutic option in the future [17].

## Conclusions

Craniopharyngiomas are a rare cause of intracranial tumor. ACP is more common overall, and most commonly occurs in children. Of the adult cases, it is rare to see this tumor diagnosed under the age of 50. We presented a rare case of ACP in a 34-year-old female. The patient underwent transsphenoidal resection with the restoration of the majority of pituitary function. This case serves to heighten our awareness of the occurrence of this type of tumor in younger adult patients and emphasizes helpful neuroimaging features, typical pathological findings, surgical techniques, and sequelae.
